# The impact of China's universal two-child policy on total, preterm, and multiple births: a nationwide interrupted time-series analysis

**DOI:** 10.1186/s12889-023-17620-5

**Published:** 2024-01-20

**Authors:** Yuehang Geng, Lin Zhuo, Rui Zhang, Houyu Zhao, Xinlin Hou, Hu Chen, Lili Liu

**Affiliations:** 1 Department of Pediatrics, Peking University First Hospital, Beijing, 100034 China; 2 Research Center of Clinical Epidemiology, Peking University Third Hospital, Beijing, 100191 China; 3 Department of Epidemiology and Biostatistics, School of Public Health, Peking University, Beijing, 100191 China; 4 Center for Medical Administration, National Health Commission of the People’s Republic of China, Beijing, 100044 China

**Keywords:** China’s two-child policy, Preterm birth, Multiple births, Interrupted time-series analysis

## Abstract

**Background:**

Although years have passed since the implementation of China’s universal two-child policy, the effectiveness of this policy remains unclear. To address this knowledge gap, we, here, assessed the impact of the two-child policy on total live births, preterm births, and multiple live births.

**Methods:**

Data identifying pregnancies resulting in at least one live birth between April 1 2013 and December 31 2018 were collected from the Hospital Quality Monitoring System database. Using an interrupted time-series analysis, we estimated immediate level changes and long-term trends in total, preterm (birth before 37 weeks’ gestation), and multiple live births that had occurred after July 2016, when the universal two-child policy had taken effect.

**Results:**

A total of 8,273,622 live births were reported during the study time frame. The number of live births (*p* = 0.277), preterm births (*p* = 0.052), and multiple births (*p* = 0.856) per month slightly increased immediately after July 2016, but these increases did not meet statistical significance. Further, all three outcomes showed a significant downward trend that lasted until the end of 2018 (*p* < 0.0001 for all). Among all live births, the percentage of preterm births remained stable (*p* = 0.101), while the percentage of multiple live births that were preterm significantly increased (trend change = 0.21% per month, 95% CI 0.14 to 0.28, *p* < 0.0001). The percentage of live multiple births among all live births significantly decreased (*p* for trend = 0.0039).

**Conclusions:**

Overall, our data reveal a transient baby boom, as well as an increase in the proportion of live multiple births that were preterm, after China’s two-child policy took effect. The latter should be noted by healthcare professionals due to the high risk of complications and special medical care required by preterm babies.

## Key messages

• Eight years have passed since the implementation of China’s universal two-child policy in October 2015, and a further three-child policy was announced in August 2021.

• The two-child policy led to a temporary “baby boom”, but the overall total number of live births declined thereafter.

• Among all live births, the percentage of preterm births remained stable, while the percentage of multiple live births that were preterm increased. This should be noted by healthcare professional due to the high risk of complications and special medical care required by preterm babies.

## Introduction

To tackle the concurrent issues of a rapidly aging population and a declining birth rate in China, a universal two-child policy was passed by the Chinese government in October 2015 and took effect on July 2016, nine months after policy introduction. The implementation of the universal two-child policy marked the end of the one-child policy, which dated back to 1982. To-date, the effect of the universal two-child policy on the number of live births and birth-related outcomes, such as preterm births, remains unclear. Indeed, the few studies that have investigated this area by leveraging either national or local databases have yielded conflicting results [1–3]. Elucidation of the effect of the universal two-child policy on birth-related outcomes is crucial for maintaining a robust healthcare system as well as is needed to predict upcoming unmet medical needs. For instance, when compared with term babies, preterm babies are at increased risk of short- and long-term adverse outcomes, including respiratory distress syndrome, necrotizing enterocolitis, bronchopulmonary dysplasia, retinopathy of prematurity periventricular leukomalacia, and other neurodevelopmental sequelae [4, 5].

Contemporary data on the number and trends in preterm births may help identify and predict unmet medical needs in this healthcare field, as well as guide expectations and healthcare preparations that may result from the recent implementation of China’s three-child policy on August 20, 2021. We, thus, investigated the effects of the universal two-child policy on total, preterm, and multiple births by using national inpatient electronic health records from April 1, 2013, and December 31, 2018, from The Hospital Quality Monitoring System database.

## Methods

### Data source

We leveraged data from the Hospital Quality Monitoring System (HQMS) database, a national electronic inpatient medical record system established in 2013 under the authority of the National Health Commission of the People's Republic of China. The database harbors information collected from 1861 tertiary hospitals spread throughout the 31 provinces in mainland China, and thus represents 52.3% of all tertiary hospitals within China. The database, which contains records from over 8 million live births during our 2013–2018 study period, routinely collects data from standardized inpatient discharge records, including patient demographics, complete discharge diagnoses, procedures, operations, and outcomes. For our study, we were granted, by the National Health Commission of the People's Republic of China, access to all records of live births with complete information including gestational age, birth weight, and single/multiple birth. We did not access any identifying personal information, such as full name, citizen's identification number, and contact information. Diagnoses are uploaded as clinical terms in Chinese and coded according to the International Statistical Classification of Diseases and Related Health Problems 10th Revision (ICD-10) by certified medical coders within each hospital. All data submitted to the HQMS database are inspected daily by medical specialists of the National Health Commission before release to ensure their completeness, accuracy, and consistency. If any aspect of data quality fails to meet these standards, all data submitted by the hospital are returned, and further review and resubmission are required. Due to these rigorous quality control measures, the HQMS database is being increasingly leveraged as an important tool for evaluating healthcare quality and changes in major health issues in China [6, 7] for the purpose of helping to inform and improve China's healthcare system.

Our study has been approved by the Ethics Committee of Peking University First Hospital (No.2021–271) and has been registered on the Chinese Clinical Trial Registry (https://www.chictr.org.cn), registry number: ChiCTR2000039164 (20/10/2020). All methods were performed following the relevant guidelines and regulations.

### Identification of study outcomes

To identify all pregnancies resulting in live births born between April 2013 and December 2018, we used the free text entry search and the following three sets of ICD-10 codes: the Beijing version 4.0, national standard version 1.0, and national clinical version 1.0. All three codes have been used in previous studies [7]. When discrepancies occurred among different ICD-10 codes and free text searches, two medical specialists reviewed the records and determined whether the record should be included through consensus. Records for which no agreement could be reached were excluded. This same search method was used to identify pregnancies resulting in preterm births and/or multiple births.

### Statistical analysis

The total number of live births was defined as the number of live births each month during the study period. The numbers of preterm and multiple births were extracted after searching by diagnoses according to the methods mentioned above. Monthly percentages of preterm and multiple births among all live births were then calculated. Finally, the proportions of preterm births and multiple births were calculated by dividing the absolute monthly preterm birth/multiple birth ratio by the total monthly live birth quantity.

To visualize the overall pattern and trend across the study period, we generated graphic displays of the numbers of total, preterm, and multiple births. Further, we used segmented regression analysis of interrupted time series (ITS) analysis [8–10] to estimate the level and change in the monthly number of total, preterm, and multiple births across the study period, as well as after China's universal two-child policy had begun to take effect on July 2016. A standard segmented regression model was established as

$${\text{Y}}t=\upbeta 0+\upbeta 1\times {\text{mont}}ht+\upbeta 2\times {\text{policy}}t+\upbeta 3\times {\text{month}}\_{\text{after}}\_{\text{policy}}t+\upbeta 4\times season\_{\text{dummy}}+ \varepsilon t$$

For each ITS analysis, *Y*_*t*_ represents the outcome: the monthly numbers of total, preterm, and multiple births, as well as the monthly proportions of preterm and multiple births. *Month* indicates the time in months from the start of the observation period (coded as 1–69, corresponding to the months from April 2013 to December 2018). *Policy*_*t*_ represents the status of the universal two-child policy (coded as 0 before July 2016 and 1 thereafter). *Month_after_policy* indicates the months after the policy has taken effect (coded as 0 before July 2016 and as 1–30 for months from July 2016 to December 2018). *Season_dummy* includes a set of 3 indicators representing the four calendar seasons (spring: Mar-May, summer: Jun-Aug, autumn: Sept-Nov, winter: Dec-Feb within each year). *εt* is an estimate of the random error at a given time.

Using this model, we aimed to estimate, for each outcome, the regression coefficient of *policy* (β2) and *month_after_policy* (β3), which can be interpreted as the change in the mean level immediately after the policy took effect and the change in the trend between the baseline period and the period after the policy took effect, respectively. The Durbin-Watson statistic test was applied for serial autocorrelation in the regression models.

Statistical analyses were conducted using SAS (version 9.0). A *p*-value of < 0.05 indicated significant difference among compared groups.

## Results

### Descriptive analyses

**Fig. 1:**
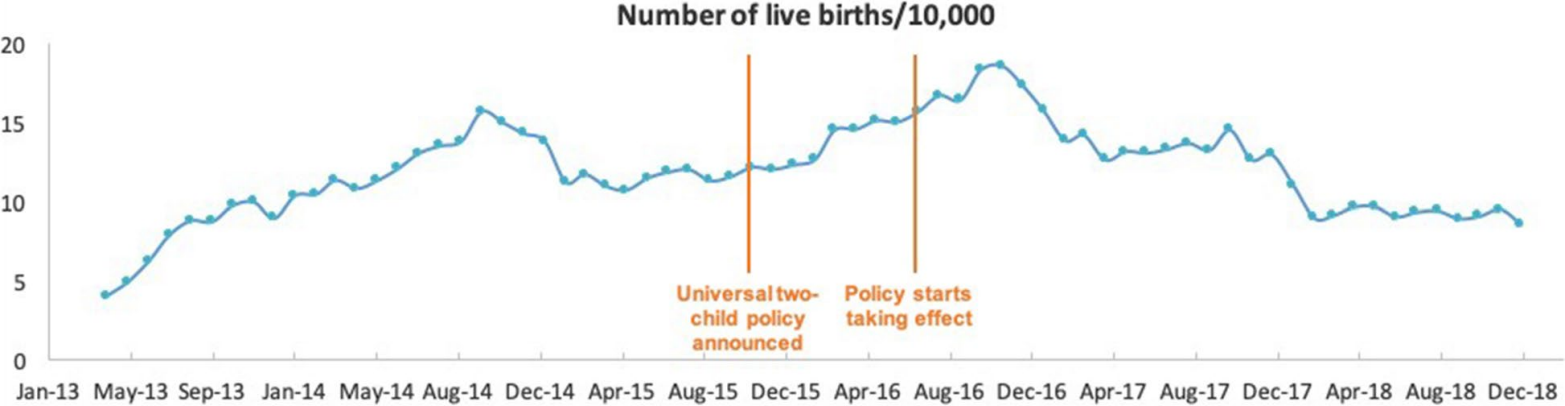
Monthly number of total live births recorded in the HQMS database from April 2013 to December 2018

From April 2013 to December 2018, a total of 8,273,622 live births were reported in the HQMS database. Strong seasonal fluctuations were observed, with the number of live births in winter months higher than that of other months (*p* < 0.0001) (Fig. 1). For each calendar year between 2014 to 2018, the annual total live births reported in the database were 1.52, 1.41, 1.88, 1.63, and 1.13 million. Among these four years, live births peaked in 2016, which corresponded to when the universal two-child policy was anticipated to take effect. After 2016, we observed a gradual decrease in live births to levels lower than those in 2014.

**Fig. 2:**
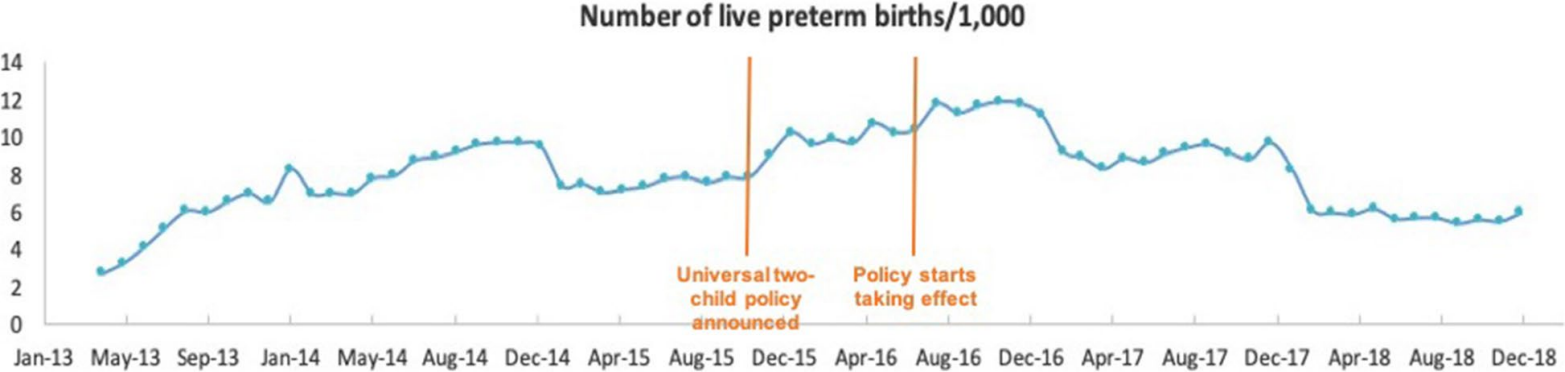
Monthly number of live preterm births recorded in the HQMS database from April 2013 to December 2018

**Fig. 3:**
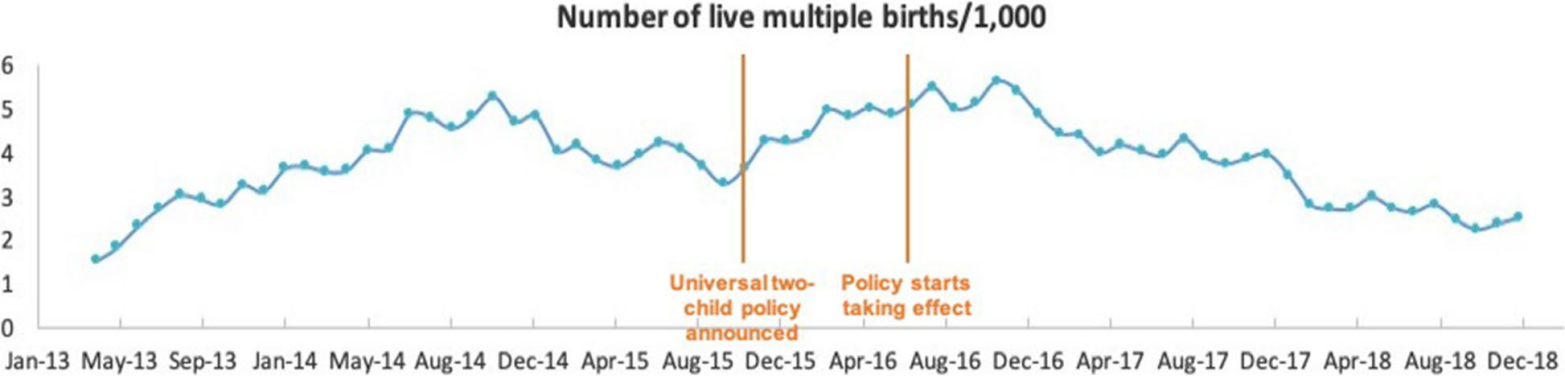
Monthly number of live multiple births recorded in the HQMS database from April 2013 to December 2018

**Fig. 4:**
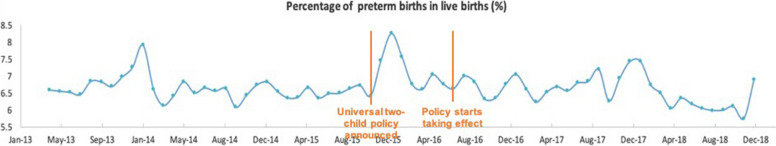
Monthly percentage of preterm births among all live births recorded in the HQMS database from April 2013 to December 2018

**Fig. 5:**
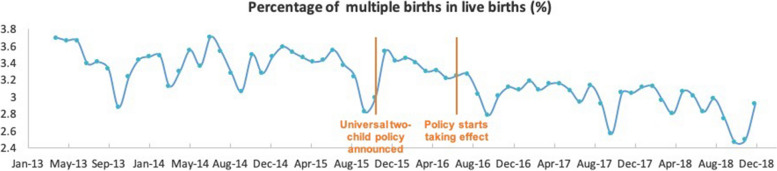
Monthly percentage of multiple births among all live births recorded in the HQMS database from April 2013 to December 2018

**Fig. 6:**
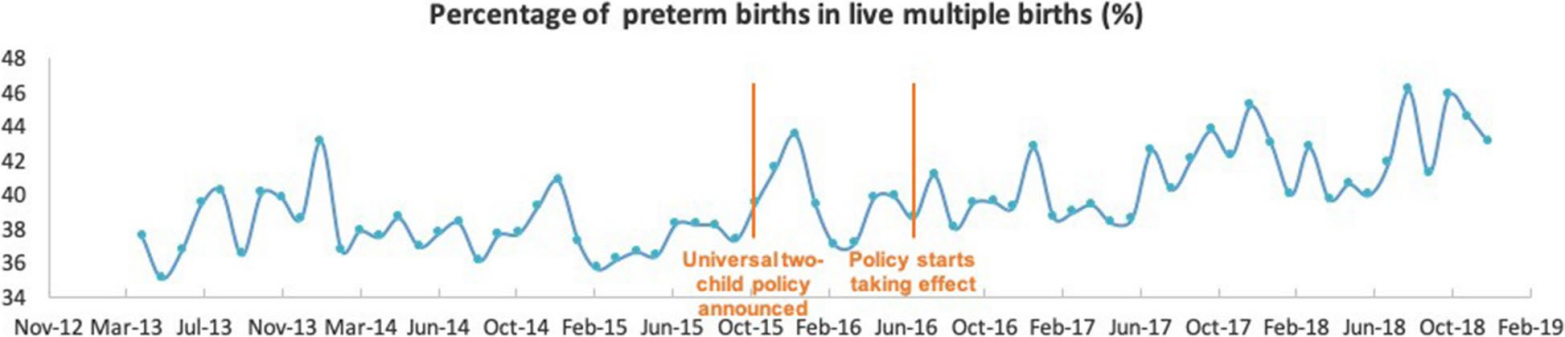
Monthly percentage of preterm births among all live multiple births recorded in the HQMS database from April 2013 to December 2018

Among all live births during the study period, 550,863 (6.66%) were preterm. On visual inspection, the overall trend and fluctuations in the number of preterm live births were similar to all live births within our study period (Fig. 2). Analysis of the monthly percentage of preterm births (Fig. 3) showed no apparent change in level or trend, averaging 6.61%, 6.60%, 6.89%, 6.75%, and 6.32% for each calendar year from 2014 to 2018. We observed similar patterns for multiple live births as for total and preterm live births per month (Fig. 4). However, the monthly percentage of live multiple births was observed to decrease after July 2016 (Fig. 5), averaging 3.38%, 3.36%, 3.21%, 3.02%, and 2.87% for each calendar year from 2014 to 2018. Although the overall percentage of multiple births trended downward post-July 2016, we observed an upward trend in the percentage of preterm births among live multiple births after July 2016 (Fig. 6). Specifically, average monthly percentages of preterm births among live multiple births were 38.1% and 38.0% in 2014 and 2015, respectively, and 39.0%, 41.1%, and 42.4%, in 2016, 2017, and 2018, respectively.

### Interrupted time-series analysis: monthly numbers and percentages

#### Total live births

In 2014, the monthly number of live births recorded in the HQMS database fluctuated around the mean of 95,600, with a gradual increasing trend observed across study years at a slope of 0.19. After July 2016, we observed an increasing trend in the monthly number of live births, with a level change of 12,859 (95% confidence interval (CI) –12,300 to 38,100) (*p* = 0.277). However, a significant decrease in the monthly number of live births (change = –4,552 live births per month, 95% CI –5.830 to –3,720, *p* < 0.0001) was observed immediately following this transient moderate increase (Table 1).

**Table 1:** Impacts of the two-child policy on number and percentage of total, preterm, and multiple births

Outcome	Baseline level	Level change			Trend change		
		Estimate	95% CI	*p* value	Estimate	95% CI	*p* value
Number of live births	95,600	12,859	-12,300 to 38,100	0.277	-4,552	-5.830 to -3,720	** < 0.0001**
Number of preterm births	6,109	1,623	13 to 3,230	0.052	-304	-361 to -247	** < 0.0001**
Number of multiple births	2,860	-84.7	-995 to 825	0.856	-165	-198 to -132	** < 0.0001**
Percentage of preterm births in live births (%)	6.76	0.49	-0.08 to 1.06	0.101	-0.0071	-0.03 to 0.02	0.5939
Percentage of multiple births in live births (%)	3.22	-0.19	-0.35 to -0.04	**0.016**	-0.011	-0.02 to -0.003	**0.0039**
Percentage of preterm births in multiple births (%)	38.6	1.41	-0.14 to 2.96	0.0794	0.21	0.14 to 0.28	** < 0.0001**

#### Preterm live births

We recorded 6,109 live preterm births in July 2016, with numbers of monthly live preterm births steadily increasing over the study years at a slope of 0.1. The number of monthly preterm live births immediately rose after July 2016 (level change = 1623, 95% CI: 13 to 3230, *p* = 0.052) and significant decreased after this date (change = –304 live preterm births per month, 95% CI –361 to –247, *p* < 0.0001). Analysis of the monthly proportion of preterm live births among all live births revealed that preterm live births in 2014 accounted for 6.76% of all live births, with no significant change in level or trend occurring after July 2016 (0.49%, 95% CI: –0.08 to 1.06, *p* = 0.101; trend change = –0.0071% per month, 95% CI: –0.03 to 0.02, *p* = 0.5939).

#### Multiple live births

We identified 2860 monthly live multiple births in 2014, with numbers increasing gradually over the study period at a slope of 0.068. After July 2016, no significant change in monthly live multiple births was immediately observed (level change = –84.7, 95% CI: 995 to 825, *p* = 0.856), but a significant decrease was apparent thereafter (trend change = –165 births per month, 95% CI: –198 to –132, *p* < 0.0001) (Table 1). In 2014, the number of live multiple births accounted for 3.22% of all live births. After July 2016, however, both an immediate significant decrease in percentage and a further significant downward trend were observed (level change = –0.19%, 95% CI: –0.35 to –0.04, *p* = 0.016; trend change = –0.011% per month, 95% CI: –0.02 to –0.003, *p* = 0.0039). Analysis of the percentage of live preterm multiple births among all live multiple births before and after July 2016 revealed that, in 2014, the percentage of live preterm multiple births among all live multiple births was approximately 38.6%, with no significant slope up to July 2016. However, after July 2016, an immediate moderate (1.41%) increase in the percentage of live preterm multiple births among all live multiple births was observed (95% CI: –0.14 to 2.96, *p* = 0.0794), followed by a significant upward trend (trend change = 0.21% per month, 95% CI: 0.14 to 0.28, *p* < 0.0001).

## Discussion

In this large nationwide study using data from 2014–2018, we found a clear correlation between the implementation of the universal two-child policy in October 2015 and the ‘baby boom’ observed around July 2016. This transient increase in live births was followed by a noticeable decline in the following two calendar years. Our data revealed an annual increase in the percentage of preterm births among live multiple births pre- and post-2016, whereas the number of all live births remained stable and the percentage of live multiple births among all live birth declined across this period of time. Further, the ITS ‘monthly’ analyses showed these trends to occur pre- and post-July 2016, during which the two-child policy was considered to have taken effect.

Among the other large population-based studies on this topic, a recent interrogation of county-level monthly aggregated data (CMAD) and individual-level delivery information records (IDIR) –both compiled according to regulations issued by the National Health Commission of China– also reported a rise in live births after when the two-child policy had taken effect, with increases greatest among multiparous mothers, but did not report a decline in live births in 2017, the last year of their 2014–2017 study period [11]. Further, this study did not uncover any change in the percentage of preterm births over their study period [11], which is consistent with our findings. However, a study leveraging data from China's National Maternal Near Miss Surveillance System (NMNMSS) uncovered a rise in preterm birth rates from 5.9% in 2012 to 6.4% in 2018 [2], which is, albeit, still lower than that observed during most years in our study. The NMNMSS includes data from 438 health facilities at the county level or higher, across 326 urban districts and rural counties in 30 provinces, while the HQMS database contains data from 1861 tertiary hospitals in 31 provinces. Pregnancy cases treated at tertiary hospitals are, in general, more complicated than those at primary/secondary hospitals, which may explain the higher overall percentage of preterm births observed in our study.

The baby boom observed in 2016 indicated the acceptance of the two-child policy introduced in China in July of that year, which enabled parents to legally have more than one child. The decline in the subsequent two calendar years was also intuitive, as the majority of parents, having already reached their maximum of two children, were unable, by law at the time, to have more children. Interestingly, our data show a clear decrease in both levels and trends in the percentage of multiple births among all live births. The reason for this decrease is unclear, as one would expect the increased uptake of assistive reproductive technology (ART) to enable an increased intake of multiple births. Additional studies are needed to further investigate this trend, as studies on the incidence of multiple births in China are scarce and have been published before the implementation of the two-child policy.

Our study uncovered a concerning increase in the percentage of preterm births among all live multiple births. Contributing factors may include advanced maternal age and ART. Studies leveraging data from provincial or national databases have reported an increase in advanced maternal age (≥ 35 years old) from 2012–2018, as the two-child policy especially encouraged pregnancies in multiparous mothers [2, 11, 12]. The reported proportion of births to “older” mothers increased from 7–8% before the policy to 13–15% in 2018. Advanced maternal age has increased the demand of ART in China, and ART has been associated with preterm and multiple gestations [13, 14]. Further studies are needed to determine causality, since many other maternal and fetal characteristics are associated with preterm and/or multiple birth, including maternal sociodemographic, nutritional, psychological, behavioral characteristics, as well as biological and genetic factors. However, regardless of the etiology of this observed trend, the increased proportion of preterm births reported among live multiple births during our study period should serve as a warning to healthcare authorities of oncoming challenges for the healthcare system, as preterm births are high risk of developing various acute and long-term complications [14–19] and will require intensive medical attention. Although preventing preterm birth remains a challenge, improving antenatal care and management for high-risk pregnancies, namely those of advanced age, with multiple gestations, would be of great value in reducing the risk of preterm birth. Efforts should include health education, increasing number and improving quality of antenatal visits, guidance in newborn care, and long-term follow up.

To the best of our knowledge, our study is the first to use both descriptive and ITS analyses to comprehensively assess the effect of the universal two-child policy on total, preterm, and multiple births using data obtained from the HQMS database, which is the single largest medical record database among tertiary hospitals in China and harbors records from approximately 10% of all live births in China within our study period. Our findings provide essential information regarding the prevention and management of preterm birth for clinical practitioners, public health professionals, and policy makers.

Our study has limitations. As a descriptive study, we were unable to establish correlation or causality of other maternal and fetal factors associated with preterm birth. While data from the HQMS database covers a wide geographical area, the volume of records within the database varies among provinces, which may have diminished the precision of our analyses. Further, the HQMS database only includes records from tertiary hospitals, which may have resulted in a selection bias towards higher-risk pregnancies and births, although the presence of such a bias would not have affected the trends observed over time.

## Conclusions

In conclusion, our nationwide study showed a correlation between the introduction of the two-child policy in China and a ‘baby boom’ after July 2016, during which this policy was anticipated to take effect. Healthcare professionals should note the higher proportion of preterm multiple births born during our study period due to the complex medical care needed by these neonates. Importantly, the methodology of our study provides a blueprint for assessing the effect of the three-child policy, introduced in China on August 20 2021, on births and birth outcomes. Indeed, we expect that findings from such a study will help uncover new, concerning patterns in birth outcomes that will warrant the attention of healthcare professionals.

## Data Availability

Data and materials used in this study are under the regulation of the National Health Commission of the People's Republic of China and are not available to the public. Yuehang Geng (email: gengyuehang@163.com) should be contacted if someone wants to request the data from this study.
